# Idiopathic Acquired Leukonychia in a 34-Year-Old Patient

**DOI:** 10.1155/2009/495809

**Published:** 2009-03-24

**Authors:** Maria Rita Bongiorno, Mario Aricò

**Affiliations:** Department of Dermatology, University of Palermo, Via del Vespro 131, 90127 Palermo, Italy

## Abstract

We present a rare case of a 34-year-old patient with persistent, progressive, acquired leukonychia totalis and partialis. Idiopathic acquired leukonychia is a rare chromatic disorder of the nail not associated with other abnormalities and discernible etiology. Our case report did not link the inheritance of leukonychia with diverse clinical syndromes. To our knowledge, only five cases of idiopathic, acquired, true total leukonychia were found in literature. This case was the sixth patient with asymptomatic idiopathic, white fingernails, and toenails without a hereditary cause.

## 1. Introduction

Leukonychia refers to whiteness of the nails which can occur either in patches or involving the entire nail [1]. The condition can be either true leukonychia, with involvement of the nail plate, or pseudoleukonychia, caused by subungual and nailbed abnormalities.

Leukonychia may be acquired or inherited. Acquired leukonychia is frequently associated with trauma, drugs such as chemotherapeutic agents, [2] systemic or local infections—such as typhoid fever, hepatic cirrhosis, ulcerative colitis or leprosy, hypocalcaemia, and, very commonly, minor trauma. True leukonychia can be inherited as an isolated condition or as one of several other reported syndromes. There are a number of autosomal, dominantly inherited leukonychia syndromes, including leukonychia totalis, in which leukonychia occurs in combination with kidney stone and sebaceous cysts [3, 4], as well as leukonychia, with sensory-neural deafness and knuckle pads, known as the Bart-Pumphrey syndrome [5, 6] (Table 1). 

Classification of the so-called true leukonychia may also be based on the distribution of white blotches, known as leukonychia punctata, leukonychia striata, leukonychia partialis, or leukonychia totalis. Idiopathic true leukonychia is a much rarer condition, with only a few reported [7–11] cases. The following is the case history of a man with persistent, progressive idiopathic leukonychia.

## 2. Case Presentation

The 34-year-old male patient was admitted in the Department of Dermatology, University of Palermo (Italy), complaining of color changes on the nails. The symptoms were observed synchronously and had apparently been present for 11 years. When the patient was 23, he developed simultaneous leuconychia partialis of both the fingers and toenails. There was no evidence of atopy, lichen planus, alopecia areata, or psoriasis.

**Figure 1:**
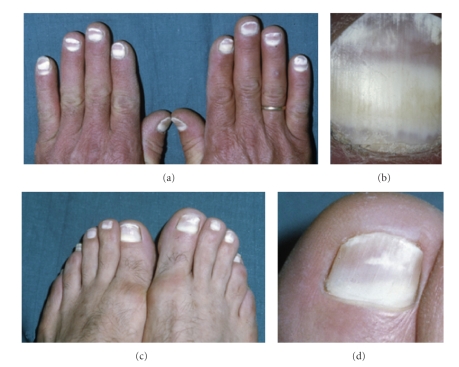
(a)-(b) Both hands had opaque nail plates characteristic of leukonychia totalis. (c)-(d) The patient toenails exhibited leukonychia totalis and partialis.

The patient was without a previous medical history. He reported not having taken any drug specific and not to be exposed to chemical agents. He also had no peripheral neurovascular disorders. On examination, striata and total leukonychia of all twenty nails was found (Figure 1). Further clinical examination revealed soft nails with slow growth, without other cutaneous or visceral abnormalities.

A progression over the years from leuconychia partialis to leuconychia totalis was demonstrated. On examination, the patient had no central nervous system, eye, ear, hair, teeth, or skin abnormalities.

There was no family history of nail disease, the patient was born to nonconsanguineous parents and had two older siblings, a brother and sister, without any features of the disease. There was no family history of atopy, psoriasis, lichen planus, alopecia areata, or any other illnesses. Repeated potassium hydroxide preparations and fungal cultures of the white nails were negative.

## 3. Discussion

Leukonychia is a whitening of the nail plate. It was first described by Mees in 1919, as an associated finding in arsenic intoxication [12].

The physiologic mechanism leading to this phenomenon is not entirely clear. According to Newton's theorem, a surface appears white when it reflects the radiation of visible light. This mechanism can be proposed in explaining leukonychia. Because true leuconychia is thought to be due to abnormal matrix keratinization, with persistent parakeratosis and keratohyaline granules in the nail plate, parakeratosis and dissociation of the keratin bundles may play a role in the modification of the solar light reflection by the ungueal plates.

Several studies have provided evidence for the association of total leukonychia with diverse clinical syndromes, including leukonychia with palmoplantar keratoderma and atrophic fibrosis, pili torti [13], congenital hypoparathyroidism, hypoparathyroidism, onychorrhexis, and cataracts and the LEOPARD syndrome [1]. Total leukonychia has also been associated with peptic ulcer disease and cholelithiasis as well as with keratoderma and hypotrichosis [14].

Our case report did not link the inheritance of leukonychia with any of the above-mentioned syndromes. To our knowledge, only five cases of idiopathic, acquired, true total leukonychia were found in literature (Table 2). This case was the sixth patient with asymptomatic idiopathic, white fingernails and toenails without a hereditary cause.

## Figures and Tables

**Table 1:** Classification of true leukonychia.

(A) Hereditary
* *(1) Isolated
* *(2) Associated with
* * * *(a) Bart-Pumphrey syndrome
* * * *(b) Bauer syndrome
* * * *(c) Heimler syndrome
* * * *(d) Knuckle pads-leukonychia-deafness syndrome
* * * *(e) Keratoderma, hypotrichosis and leukonychia totalis
* * * * * *syndrome
* * * *(f) Lowry-Wood syndrome
* * * *(g) FLOTCH syndrome
* * * *(h) Congenital keratosis palmaris et plantaris, deafness
* * * * * *and total leukonychia
* * * *(i) LEOPARD syndrome
(B) Acquired
* *(1) Idiopathic
* *(2) Associated with
* * * *(a) Trauma
* * * *(b) Drugs
* * * *(c) Systemic infections
* * * *(d) Local infections
* * * *(e) Inflammatory disease.

**Table 2:** True acquired idiopathic leukonychia.

Authors	Presentations	Active disease (years)
Claudel et al. [8]	Leukonychia totalis and partialis	2
Grossman et al. [1]	Leukonychia partialis to a combined partialis and totalis	3
Stewart et al. [7]	Leukonychia totalis and partialis	Unrelated
Park et al. [10]	Leuconychia partialis to leuconychia totalis	13
Butterworth [11]	Leukonychia totalis and partialis	Unrelated
Our case	Leuconychia partialis to leuconychia totalis	11
